# Content Analysis of Digital Archives Contributes to the Historical Distribution and Folk Knowledge of the Highly Toxic *Cicuta virosa* L. in Hungary

**DOI:** 10.3390/plants14030315

**Published:** 2025-01-21

**Authors:** Szabolcs Kis, Attila Molnár V.

**Affiliations:** HUN-REN–UD Conservation Biology Research Group, Department of Botany, University of Debrecen, Egyetem sq. 1., H-4032 Debrecen, Hungary

**Keywords:** Apiaceae, Central Europe, children’s snack, ethnoveterinary, habitat loss, local ecological knowledge, Pannonian ecoregion, poisonous plants, wetlands

## Abstract

The northern water hemlock is an endangered species that has been severely diminished in Hungary due to water regulation and river control in the 18th and 19th centuries. We collected data on this highly toxic plant from Hungary using archival sources, including digitized databases of daily and weekly newspapers and books. By exploring historical digital archives, we identified 88 locatable occurrence records spanning 65 flora mapping grids, 52 of which represent new additions to its known distribution. Between 1721 and 1943, a total of 103 records were found relating to its vernacular names. The most widespread names were *csomorika* (predominantly used in the Berettyó-Sárrét and Hortobágy regions), *mételytorzsa* (Rétköz, Taktaköz, Ecsedi-láp), and *Kónyi gyökér* (Fertő-Hanság region). Human poisonings caused by this species were primarily due to confusion with parsley, celery, and, less frequently, parsnips or carrots, occasionally resulting in the deaths of entire families. Children, in particular, were at risk when they accidentally consumed it raw as a snack. There have also been instances of intentional homicidal use and unintentional fatalities associated with its ethnomedicinal application. The plant was primarily used to treat scrofula (a form of tuberculosis-induced lymphoma) and for abortion.

## 1. Introduction

Human-induced modifications to the landscape are a significant driver of biodiversity loss [1]. Such activities include the ploughing of grasslands, deforestation, and wetland drainage. A particularly noteworthy example is the profound transformation of the soil, hydrological, climatic, and ecological conditions of the Hungarian Great Plain resulting from river regulation [2]. Prior to the implementation of river regulation and drainage in the 18th century, a substantial portion of the Pannonian Basin was periodically or permanently covered by water [3]. In the valley of the River Tisza, the largest river regulation project in Europe led to the destruction of approximately 38,000 km^2^ of wetlands. This significant landscape alteration resulted in considerable changes to both vegetation and fauna, including a dramatic 83% reduction in reedbed areas and the complete disappearance of floating islands [4].

As a result, this extensive landscape transformation has left us with limited information regarding the flora and vegetation of the former wetland habitats, further exacerbating the knowledge gap created by the river regulation efforts. To address this deficiency, the exploration of historical digital archives, which have previously proven effective in studying past wetland grazing [4], emerges as a promising solution.

For this purpose, we selected the highly toxic *Cicuta virosa* L. as a model species, hypothesizing that its toxicity may have played a significant role in folk life, a role that may be reflected in historical, printed sources contained within digital archives.

Northern water hemlock (*Cicuta virosa*) is the only European representative of the genus. It is a circumpolar species, distributed across Eurasia (from Western and Central Europe to Siberia, and the Korean Peninsula) and northwestern North America [5,6,7,8].

**Figure 1:**
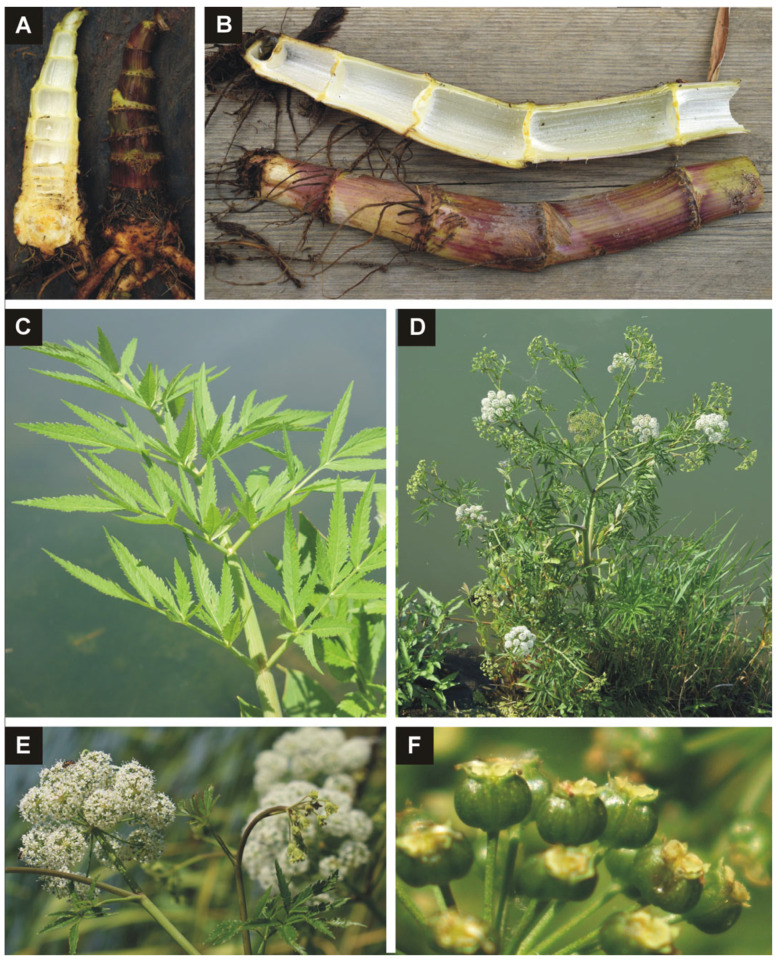
Morphological characteristics of *Cicuta virosa*. (**A**) Rhizome resembling celery and its longitudinal section with leaking yellowish, oily liquid; (**B**) hollow, articulate, generative stem with nodular adventitious roots and its longitudinal section; (**C**) leaf; (**D**) flowering individual; (**E**) inflorescence; (**F**) unripe fruits. Photo credits (**A**,**B**,**D**) by A. Molnár V.; (**C**,**E**,**F**) by Sz. Kis.

*Cicuta virosa* is a hemicryptophyte (hydro-helophytic) species. Its rootstock is multichambered, containing a yellowish oily liquid (Figure 1A) and emitting a characteristic smell of raw parsnip. The stem is thick and hollow (Figure 1B), and the petioles are inflated and hollow. The leaves are alternate, pinnately compound (two or three times) (Figure 1C), and may reach 30–120 cm in length. The leaflets are elliptic–lanceolate with acutely serrate margins. The generative shoots are hollow with clusters of foliage (Figure 1B). The umbellate inflorescence contains clusters of 30–50 flowers with white petals (Figure 1E). The schizocarp fruits are approximately 2 mm long and 3 mm wide, with a much longer pedicel (Figure 1F) [9,10]: 352.

The entire plant is highly toxic, with its primary active compounds being C-17 polyacetylene-type substances that induce respiratory paralysis and death through GABAA receptor antagonism [11] and also inhibit potassium channels [12]. The most notable of these toxins is cicutoxin [13,14], which has historically been responsible for fatal poisonings among both humans and livestock [15]. The cicutoxin content in fresh plant material is approximately 1.5%, and in dried material, it can reach 3.5% [10]. It also contains 11 other C-17 polyacetylene derivatives, the most important of which are oenanthotoxin, cicutol, falcarindiol, and isocicutol [14].

Currently, *Cicuta virosa* is listed as endangered (EN) in Hungary [16], the Czech Republic [17], and South Korea [8], and as vulnerable (VU) in Slovakia [18]. In Baden-Württemberg (Germany), the species is highly endangered, with its decline largely attributed to the conversion and overuse of water bodies [10].

Our work was based on the two following assumptions: (i) *Cicuta virosa* may have been much more widespread in Hungary before the river regulation and drainage works of the 19th century; (ii) reports of human and livestock poisoning cases may have appeared in archival, non-scientific sources, such as newspapers, magazines, and periodicals of the time, and these contemporary records may provide valuable insights into the former distribution and local knowledge related to the species.

## 2. Results

**Figure 2:**
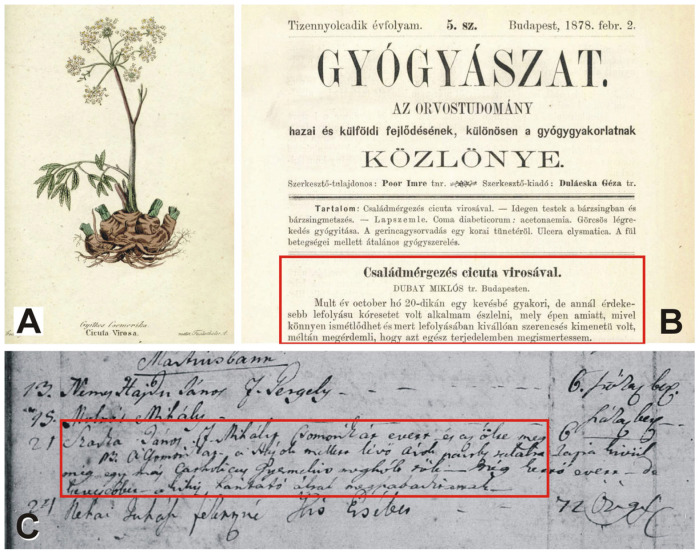
*Cicuta virosa* in archival sources. (**A**) Illustration of the species in the work of Á. Kubinyi [19] published in 1842; (**B**) “Family poisoning with *Cicuta virosa*”: news on the cover of the journal *Gyógyászat* in 1878: “On the 20th of October last year, I had the opportunity to observe a case of a rather uncommon yet intriguing course of illness, which, precisely because it could easily recur and had an exceptionally fortunate outcome, rightfully deserves to be presented in its entirety” [20]; (**C**) death certificate of a 6-year-old boy who consumed *csomorika* in 1803, Szeghalom, Eastern Hungary: “The son of János Szarka, Mihály, ate the csomorika which caused his death. He found the csomorika by the bank of the ditch near their house, and in addition to him, one Catholic children perished, while two others ate less and were saved by means of vomiting.”. (**C**): photographed by J. Éliás.

The studied Hungarian historical sources contain a wealth of information on *Cicuta virosa* (Figure 2).

### 2.1. Historical Distribution of the Species

**Figure 3:**
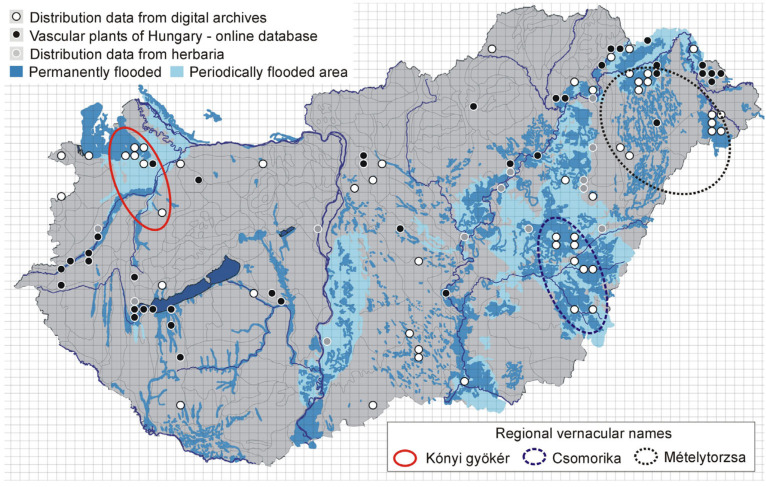
Three most common regionally used vernacular names and complete distribution of *Cicuta virosa* in Hungary, overlaid with the pattern of inundation. Areas that were permanently or temporarily covered with water in Hungary prior to river regulation are distinguished (according to the Hungarian Hydrographic Institute [3]). Full circles represent distribution data obtained from the *Vascular Plants of Hungary* online database [21].

Currently, *Cicuta virosa* is a rare and endangered species in Hungary. The Vascular Plants of Hungary online database [21] documents its occurrence in 39 flora mapping grids. Through exploration of the historical digital archives, we identified 88 locatable occurrence records covering 65 flora mapping grids, 52 of which represent additional distribution points (Figure 3). By overlaying the hydrographic depiction of the Pannonian Basin’s pre-regulation conditions onto the distribution data map derived from digital archives, it becomes evident that the recently identified distribution points correspond to locations that were once covered by extensive wetlands, characterized by periodic or permanent flooding. From this, we can infer that nearly half of the previous *Cicuta virosa* populations have been lost due to the river regulation and drainage projects in the Pannonian Basin.

### 2.2. Vernacular Names of Cicuta virosa

**Figure 4:**
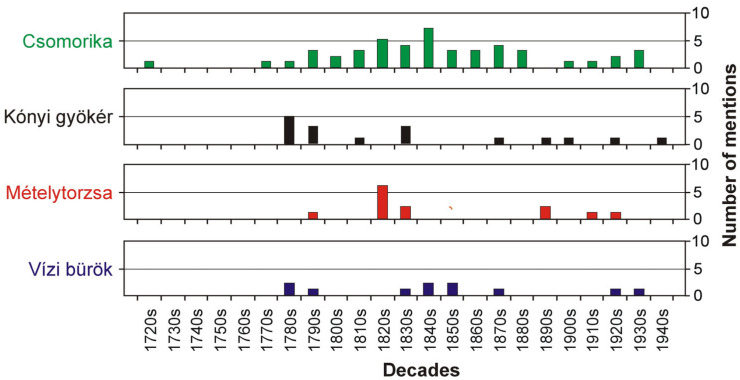
Temporal pattern of usage of the most common vernacular names for *Cicuta virosa*. The bars represent the number of mentions for each name (including variants) across decades in the studied literature.

Between 1721 and 1943, a total of 103 records were found concerning the Hungarian vernacular names of the studied species (). The most widespread names and their variants, along with their localization, sources, and brief explanations, are listed in Table 1. The most common name, *csomorika*, is still used in official Hungarian nomenclature to designate the species and has been known since 1578 (*czomorika*) [22]. The four most common names were found in the sources studied from at least the late 18th to the early 20th century (Figure 4).

### 2.3. Incidents of Poisoning

Diószegi Sámuel, a Reformed preacher and renowned botanist, wrote the following about *Cicuta virosa*: “The csomorika, among all plants, is the most wicked poison, particularly its root, which can kill both humans and livestock in a short period” [23]: 195.

#### 2.3.1. Human Poisoning

Historical records indicate the consumption of approximately 60 wild plant species among Hungarian ethnic groups [24]. At least five species from the Apiaceae family were commonly consumed in Hungary as soup, a spice, refreshing drinks, or children’s snacks (*Anthriscus cerefolium* (L.) Hoffm., *Daucus carota* L., *Heracleum sphondylium* L., *Pastinaca sativa* L., *Chaerophyllum bulbosum* L.; [24,25]). Available data show that *Cicuta virosa* was most commonly mistaken for parsley (seven cases) or celery (three cases), and less frequently for parsnip or carrot. In a detailed incident, an entire family was poisoned in 1878 by the root of *Cicuta virosa* mistaken for celery purchased at the local market [20]. In 1880, *csomorika* was cooked in soup, and the family later succumbed to poisoning [26]. “It has caused countless household poisonings, with its beautiful white umbellate flowers, leaves resembling celery, hollow roots mistaken for parsley, or its seeds, similar to aniseed” [27]: 108.

Many cases of poisoning have occurred among children who mistook it for other edible roots, often resulting in death [28]: 203. In 1787, a schoolboy in Losonc (now Lučenec, Slovakia) died after consuming the root, mistaking it for a carrot [19]. In 1796, three children on the outskirts of Pest died after consuming *Cicuta virosa* [29]. According to the Reformed Church registry in Szeghalom (March 1803), four children consumed a *csomorika* found on the banks of a ditch; two died, while two were saved after vomiting [30]. In 1816, a prayer was offered for a child who had eaten *csomorika* [31]: 201. In 1842, two boys and six girls ate *csomorika* root mistakenly identified as parsnip, and some died soon after [19]. Additionally, children used the hollow stems to make whistles, unwittingly endangering themselves by putting them in their mouths [32].

In 1722, during a witch trial in Békés County, a man was killed by his wife, who prepared bath water with *Cicuta virosa* [33,34]: 22. The famous physician and naturalist János Földi wrote, “Many people still prepare murderous poison from it”. He believed that instead of marking cultivation sites with prohibition signs, people should be informed about the plant’s medicinal properties [29]. In a case in Dévaványa, a woman cooked *csomorika* leaves in a stew for her alcohol addict husband, leading to his death by evening [35]. The plant is also mentioned in a darkly humorous folk song, ‘Ki az urát nem szereti, Csomorikát főzzön neki, Ha ma vele megeteti, Még estvére kiteríti’ (“If she does not love her husband, cook him *Csomorika*. If she feeds him today, he will be dead by night”) [36].

Although *Cicuta virosa* is not mentioned in Fäller’s (1943) comprehensive work [37], historical sources suggest its use in folk medicine, primarily against tuberculosis (lymphatic gland inflammation) [38] and rheumatism [39]: 6. In Kiskunmaja, Kígyós, and Szank, it was used as an abortifacient. The root was washed and carefully inserted into the uterine cavity; it induced miscarriage after 3 h [40]: 296. In some cases, these practices resulted in fatalities, and records of criminal proceedings initiated thereafter have been preserved. In 1868, a market vendor in Hajdúszoboszló confessed to having cured a six-year-old girl suffering from tuberculosis by preparing a decoction of *Cicuta virosa* root in milk. The child died that evening in considerable suffering [38].

#### 2.3.2. Poisoning Among Livestock

The majority of historical sources document cases of livestock mortality due to poisoning. Three examples of reports of poisonings caused by *csomorika* from different Hungarian regions during 18th century are listed in Table 2. Sometimes *Cicuta* caused significant damage; for instance, Pál Kitaibel reported that in 1799, it resulted in the death of twelve oxen near Pápa [41]. According to complaints from peasants in Szatmár County, “about one-third of the cattle are lost every four years, partly due to the *mételytorzsa*” [42]: 436.

In particular, in the Berettyó-Sárrét region (comprising the former Békés and Bihar counties), where pastures were scarce, residents grazed their animals in the marshes of the Nagy-Sárrét, where they often succumbed to poisoning. During the 18th and 19th centuries, the damage caused by *csomorika* was substantial [45]: 5. In the Hanság region, as in Berettyó-Sárrét, it caused poisoning in cattle grazing on pastures in many instances [46]. *Cicuta* could have entered the animals’ diet either through hay used as winter feed for sheep or through grazing in marshes [29]. However, sources also indicate that cattle generally consumed *csomorika* only in times of scarcity (autumn and winter) or in swampy areas where other vegetation was unavailable [47,48]. Reports of livestock poisoning from *Cicuta* in Hungary include cattle, sheep, and horses. “It has often been observed that *csomorika* kills horses, cattle, and sheep when carelessly offered to them as food” [19]. The majority of poisoned livestock were cattle (35 cases), followed by horses (six cases) and sheep (four cases). However, Diószegi [23] noted that “it is said that the leaves of *csomorika* can be safely consumed by sheep”. According to Greschik [49], larks and quails can pick the seeds of *Cicuta* without danger.

The course of poisoning is usually rapid; in cattle, it typically occurs within 5–6 h [48], a few hours [50], or even within half an hour [43]. Symptoms of poisoning include “inability to walk, muscle twitching, whitening and lividity of the tongue, cessation of mucus production (dry throat), and difficulty breathing” [50]. Kátai (1858) [51] described the effects as follows: “When consumed, it causes intense heat, pain, insatiable thirst, gastric ulceration, hiccups, spasms, dizziness, a sensation of drunkenness, convulsions, and ultimately death. After death, the abdomen swells to an enormous size, with the intestines and stomach distended due to inflammation, causing them to collapse, while the brain vessels swell with blood.” According to Kiss [46], “It is such a poisonous plant that when cattle find its leaves mixed with other grasses, they become so bloated that their skin nearly bursts open, leading to death.” Upon dissecting two steers that died from poisoning, it was reported that “the rumen was highly bloated with gases, the reticulum was dry and hard with locust leaves and root parts of the plant. The lungs were congested with blood and air was sparse. In both cases, the diaphragm was pressed tightly against the lungs. Other organs were intact. When the rumen was punctured during dissection, gas escaped with a trumpet-like rattle, similar to that of a drumstick”.

Salt was considered the most effective antidote in ethnoveterinary for poisoning by *Cicuta*. In Füzesgyarmat, “salt is considered the best antidote. Therefore, if they notice early enough that the cattle have been poisoned, they vigorously rub their tongues with salt and administer water saturated with salt” [52]. In Sárrét (Bihar county), salt was also placed into the mouth of a sheep that had consumed *csomorika*, or, in the absence of salt, the shepherd would urinate into the sheep’s mouth [53]. Tannic acid [54], milk, and fat were also mentioned as antidotes [55]: 786.

The rhizome is most commonly reported as the poisonous part of the plant (20 times), followed by the leaf (10 cases), and the stem and fruit (1–1 cases). It has also been noted that the evaporation of the plant’s leaves can cause dizziness and fainting [28,46], and that a person could faint who is lying on a pillow with a *Cicuta* underneath [56]. Kubinyi [19] suggested that the poisonous sap of the plant could contaminate water, especially in areas where the plant grows abundantly.

Considering the damage caused to livestock and human lives, it is possible that the decline in *Cicuta virosa* (Apiaceae) may have been due to its toxicity, in addition to the draining of its habitats. Diószegi [23] stated, “it is only beneficial to eradicate it wherever it is found”. Anonymous [57] noted, “*csomorika* weeds must be eradicated so that the crop of God may grow”. In some provinces, the eradication of the *csomorika* is strictly ordered [19].

## 3. Discussion

The implementation of river regulation and drainage projects has profoundly impacted wetland habitats, leading to a significant reduction in species abundance and biodiversity, primarily due to alterations in hydrological conditions [58,59]. Our analysis of historical sources indicates that *Cicuta virosa* was far more prevalent in the Pannonian Basin prior to the initiation of river regulation and drainage. Subsequent drainage efforts have led to the disappearance or substantial modification of the species’ habitats. Historical sources have provided considerable information on the vernacular names of the species, the symptoms, danger, and remedies used, and the extent of damage in cases of poisoning.

In a broader context, it is evident that plant poisonings among livestock and humans occur widely across both spatial and temporal dimensions. Numerous plant species, such as *Conium maculatum* L., *Equisetum palustre* L., *Colchicum autumnale* L., and *Datura stramonium* L., present a significant threat to grazing livestock due to their toxicity [60]. However, members of the genus *Cicuta* and related species are particularly implicated in a high number of severe poisoning cases. This has been observed in case of *Oenanthe crocata* L. in France [61] and the United Kingdom [62]. Comparable toxic effects have been associated with *Oenanthe aquatica* (L.) Poir., which has caused poisonings in grazing cattle in Sweden [63] and Poland [64].

Poisoning incidents linked to *Cicuta virosa* have been extensively documented, particularly in the northern regions of Europe [65]. Carl Linnaeus devoted considerable attention to *Cicuta virosa* in his travelogues, letters, and books [66,67]. The plant was one of 643 species studied for the food preferences of cows, goats, sheep, horses, and pigs [68]. Cases of poisoning in grazing animals, including dairy cattle, beef cattle, and ewes, continue to be reported in areas where *Cicuta virosa* is prevalent, especially in northeastern Germany [69]. Additionally, two closely related species, *Cicuta maculata* L. and *Cicuta douglasii* (DC.) Coult. & Rose, have been responsible for poisoning incidents in North America, affecting both swine and cattle. One such incident in northwestern Utah resulted in the deaths of nine Hereford cows after they ingested *Cicuta maculata* in a streamside habitat. Necropsy revealed stomach contents containing cicutoxin and two other cicutol-like compounds [70]. These observations underscore the continued relevance of plant toxicity in livestock management.

Reports of human poisonings attributed to *Cicuta virosa* have emerged in several countries, including Norway [71], The Netherlands [72], Poland [73], and Sweden [74]. Similarly, *Cicuta maculata* has been implicated in multiple human poisoning cases in North America. One tragic incident involved the fatal poisoning of a 14-year-old boy, who mistakenly identified *Cicuta maculata* as a ‘wild carrot’ [75]. Children appear to be particularly vulnerable, as evidenced by reports from various parts of the world, including Poland [73], France [76], and the United States [77]. The latter report described an incident in which 17 boys, aged 9 to 13, exhibited symptoms of poisoning. It was later revealed that five of the boys had consumed the root of *Cicuta maculata*, resulting in severe convulsions, while the remaining twelve had ingested only its leaves and flowers.

The lesson of our article is the importance of information in non-academic sources written in local languages (such as daily and weekly newspapers), whose accessibility is increasing dramatically with digitization. In particular, these sources can contribute important information about organisms that have played an important role in the life of humankind (e.g., species used as animal feed or human food or for handicraft production, and poisonous taxa). The challenges associated with processing articles written in national (non-English) languages present difficulties in preparing comprehensive scientific reviews [78]. The importance of non-English-language scientific articles in biological research has been acknowledged by several authors [79,80]. Furthermore, printed media (e.g., newspapers) and online platforms occasionally contribute to raising awareness of various issues in conservation biology [81,82].

## 4. Materials and Methods

To investigate the former distribution and local folk knowledge of *Cicuta virosa*, we conducted content analysis using the digital archives of Arcanum [83], Hungaricana [84], and Szaktárs [85]. These archives contain digitized Hungarian-language journals, magazines, dailies, weeklies, courtesy letters, and books. The following search terms were employed: the scientific names of the species (*Cicuta*, *Cicuta virosa*, *Cicuta angustifolia*, *Cicuta linearis*), the official Hungarian name of the species (*gyilkos csomorika*), and various folk names of the species (*csomorika*, *csomorika fű*, *tsomorika*, *mételytorzsa*, *méregbürök*, *vízibürök*, *kony gyökér*, *konygyökér*, *kónyi gyökér*, *kónyigyökér*). For the results obtained, the following data were recorded in an Excel spreadsheet: locality, date, folk names, poisoning incidents, symptoms, number of fatal incidents, antidotes, ethnomedicinal or ethnoveterinary uses, and plant parts consumed or used. Distribution data refer to the records that could be identified with sufficient precision to be placed in the flora mapping grids of the Central European Flora Mapping System [86] and that were not included in the Vascular Plants of Hungary online database [21] and had no data in the collections of the four most important Hungarian herbaria (BP, DE, EGR, BPU) [87,88,89,90]. The taxon does not even seem to be reported by archaeobotanical research in Hungary (e.g., see Gyulai [91]).

## 5. Conclusions

Our study has confirmed our hypothesis that historical digital archives in Hungarian contain substantial information regarding the past distribution and knowledge of the highly poisonous species *Cicuta virosa*. Thus, it can be asserted that digital archives in non-English languages hold significant potential for enhancing ethnobiological and biogeographical knowledge of organism with significant economic and/or cultural significance.

## Figures and Tables

**Table 1:** Frequency of use of various vernacular names, their geographical distribution, and brief explanations of the Hungarian terms.

Vernacular Name (Number of Mentions)	Region	Notes
*Csomorika* (22)*; Csomorika fű* (3); *Gyilkos csomorika* (19), *Mérges csomorika* (3)	Berettyó-Sárrét region, Hortobágy (E-Hungary)	*Csomorika* is the most widespread and currently used name for the plant. The specific epithets (*gyilkos*, *mérges*) refer to toxicity of the plant (killer and poisonous, respectively).
*Mételytorzsa* (9), *Métel* (1), *Métely* (3), *Mételfű* (1).	Rétköz, Taktaköz, Ecsedi-láp (NE-Hungary)	The Hungarian word *torzsa* means a thick, fleshy plant stem. The word *métely* refers to the liver fluke (*Fasciola hepatica*, Platyhelminthes), a parasite of ruminants. In a general sense, however, the word *métely* means a pathogenic, harmful organism. *Megmételyezni* = to spoil, poison, influence in a negative direction.
*Kónyi gyökér* (15), *Konyer Wurzel* (1)	Hanság, Fertő (NW-Hungary)	*Kóny* is a village in north-western Hungary, where the plant was common in the early 20th century. The word *gyökér* means root.
*Vízibürök* (6), *Vízi mérgesbürök* (2) *Méregbürök* (4)	In several regions.	The word *bürök* is usually used to designate the species *Conium maculatum*, which also belongs to the Apiaceae family and is severely poisonous too. *Cicuta* is usually distinguished from *Conium* based on their habitats (*víz* means water, *vízi* is equivalent to aquatic).

**Table 2:** Examples of sources providing ethnobotanical data on livestock poisoning from the 18th century.

Region	Original Text	English Translation	**Source**
Nagy-Sárrét	‘*A lapos és posványos hellyeken levő csomorika nevezetű fű a marhának olly veszedelmes, hogy ha azt megeszi, fél óra múlván elkelletik néki veszni. E miatt is marháinkban esztendőnként nagy kárt szenvedünk*.’	The herb, called *csomorika*, which is found in marshy places, is so dangerous to cattle that if they eat it, they are forced to die after half an hour. Because of this we also suffer a lot of damage to our cattle every year.	[43]: 331
Hajdúság	‘*Egy nyáj juhot téli időben szénával éppen olyan rét aljon étettek, ahol az ilyen Tsomorikának torzsás gyökerei bőven találtattanak. Ezt a juhok még inkább rágták, mint a szénát (minthogy ezt édességének okáért különben is szeretik). De csakhamar kezdetiének is tőle számosan ledobbanni és megdögleni*.’	In winter, a flock of sheep was fed by hay at the bottom of a meadow where the roots of such “Tsomorika” were found in abundance. The sheep like it for its sweetness and chewed it even more than hay. But it soon began to cause many of them to fall and die.	[29]
Hanság	‘*A’ Hanyságbann is valamint a’ Kónyi-tó mellett a’ Vármegye szélén, bőven találtatik az úgy nevezett Kónyi-gyökér melly olly mérges, hogy ha a’ marha meg találja enni, azonnal feldagad ’s megdöglik*.’	In Hanyság as well as near Lake Kónyi on the edge of the county, there is plenty of the so-called Kónyi root which is so poisonous that if the cattle find it to eat, it swells up and dies immediately.	[44]: 805

## Data Availability

The original contributions presented in this study are included in the Supplementary Materials. Further inquiries can be directed to the corresponding authors.

## Supplementary Materials

The following supporting information can be downloaded at: https://www.mdpi.com/article/10.3390/plants14030315/s1, Table S1: Distribution, ethnomedicinal, and ethnoveterinary information related to *Cicuta virosa* in the archival sources studied. For the complete reference list, please see the main text. References [92,93,94,95,96,97,98,99,100,101,102,103,104,105,106,107,108,109,110,111,112,113,114,115,116,117,118,119,120,121,122,123,124,125,126,127,128,129,130,131,132,133,134,135,136,137,138,139,140,141,142,143,144,145,146,147,148,149,150,151] are cited in the Supplementary Materials.
