# Supplementary material for: Content Analysis of Digital Archives Contributes to the Historical Distribution and Folk Knowledge of the Highly Toxic Cicuta virosa L. in Hungary
Source: Plants (Basel). 2025 Jan 21;14(3):315. doi: 10.3390/plants14030315 (PMC11820512; doi:10.3390/plants14030315)
Supplement: Supplementary file 1 [file plants-14-00315-s001.zip › plants-3427425-supplementary.pdf]

Table S1. Distribution, ethnomedicinal, ethnoveterinary information related to *Cicuta virosa* in the archival sources studied. For the complete reference list, please see the main text. Abbreviations used: c.: century; **Ref. (1)**: Reference; **Code (2)**: code of flora mapping grid in Central European Flora mapping system [86]; **Human pois. (3)**: Human poisoning, **+**: fatal case; **Misid. (4)**: Misidentified plant(s). A: anise, C: celery, Ca: carrot, P: parsley, Pp: parsnip; **Ethnom. (5)**: Ethnomedicinal knowledge, TCL: tuberculosis caused lymphoma , Abort: Abortifacient; **Anim. (6)**: Animal poisoning; **Plant part (7)**: Plant part(s) causing poisoning. F: Fruit, L: leaf, R: rhizome, S: stem.

Source	Ref. (1)	Location	Date	Name	Code (2)	Human pois. (3)	Misid. (4)	Ethnom.(5)	Anim. (6)	Plant part (7)	Original text
Csákabo-nyi 1960	[34]	Sarkad	1721	csomorika	9294.3	+	-	-	-		„1722. augusztus 25-én László Miklós szolgabíró maga elé rendelte a szomszédokat s a következő kérdésekkel fordult hozzájuk: »Tudják-é, gyulai Kovács Istvának miképen történt halála, s ki tudja micsodás füvekből főzött fürdőben mosta meg az urát Kovács Istvánné és azon füvek szoktak-e halált szerezni, s nevezett szerint minek hivatik azon fű?« […] Az első tanú Kecskés András volt: ^Hallotta a Kovács István szolgájától, hogy asszonya Kovács Istvánné adott egy máriást Gréczi Jánosnénak, hogy hozzon neki csomorika füvet, ezen fű felől pedig azt hallotta, hogy a mely embert megfürösztenek benne, az a halált nem kerülheti el. Azt is hallotta a szolgától a tanú, hogy Kovács Istvánné egy máriást adott neki is, hogy hozzon egérmaszlagot Aradról, de azt nem adtak, hanem hozott mercuriumot."
Bak 2000	[96]	Hanság	1773-1808	Kónyi gyökér		-	-	-	-		„A Hanságban mérges növény nő, melyet "Kónyi Gyökér"-nek hívnak. Rk templom./K Nádja elég van. Jó lovakat nevelnek, "halaik jó ízűek a Kónyi tóban, nevezetes a Kónyi mérges gyökérről".
Cseh 1989	[43]	Butsai puszta	1770	csomorika fű	8892.1	-	-	-	cattle		„1770. […] A hellységnek maleficium/a/i és kárvallás/a/i […] Bejáró marháinknak közönséges legelő mezeje igen kitsiny, és épen nem elegendő. Azértis marháink többnyire az ugaron sinlődnek őszi vetésig. Vetéskor kintelenittetűnk ezeknek számukra is tiltott mezőket szabadítani és mivel az ugarok a legelésre nem mindenkor alkalmatosak, többnyire marháink nádas és vizes réteken élődnek, a fiatal marháknak nagy veszedelmivel. Gulyabeli marhánkat és szilaj ménesünket Butsai pusztán tartyuk, a melly mivel tsak szigetekből áll, ezen levő marháink is a vizekben levő gazokon élődnek, a fiatal marháknak és sokszor az öregeknek is nagy veszedelmével. Mellyhez az is járul, hogy a lapos és posványos hellyeken levő csomorika nevezetű fű a marhának olly veszedelmes, hogy ha azt megeszi, fél óra múlván elkelletik néki veszni. E miatt is marháinkban esztendőnként nagy kárt szenvedünk."
Rácz 1794	[140]	-	1781	vízi bürök		+	-	-	-		„A vízi bürök is (cicuta aquatica) mérges, gombák is vagynak mérgesek, ezekre is, és a több eledelűl a kofáktól árulni szokott plántákra is különösen kellenne értelmes emberek által vígyáztatni."
Molnár 1783	[128]	Acsalag	1783	Kónyi gyökér	8369.1	-	-	-	-		„A fertő régi Peiso tava, mellynek vizét Galérius Tsászár a Dunába vezetteté. Úgy tetszik ez a Tsászár fertőtől egész a Rábáig. (A Rábtzát akará nevezni: mert továbbá így szóll) A Rába motsáros térségben ered Nap-kelet felé. (Az pedig a Rábtzáról kell érteni.) (Méltó volna a Hanságat, melly a temérdek vizre teritett földböl, gazos, nádós, rekettyés, ágos, fás, szenás ingoványból áll, tudós szemmel meglátogatni. Láttam azt futó félbe Atsalagnál, s a földbe tűzött náddal a föld-alól vizet szittam; de le nem merém nyelni, eszembe forgatván a Kónyi gyökér mérgét, látni helyel közzel hosszára terjedett s árnyékos vizet. Látni nagy fákat is rajta. Méltó Oslinál, s Bősárkánynál is megtekéngetni, hogy bukkaninik ki a Rábtza (ott úgy tetszik) a föld alól. De térjünk Pokokhoz vissza) A fertő vize sóos, kevés a hala. Hunnimundus Sávia Királyja Theodemirtől a Gottusok Királyjának Valamirnek Attyafiától itt talpig győzetett. Volfnál azon tó-mellett kénköves a víz. Kis-Mártonban ditsőséges palotával bír az Eszterházi Hertzeg."
		Osli-hany			8368.2	-	-	-	-		
		Bősárkány			8269.3	-	-	-	-		
Bél in Reisz 2002	[97]	Hanság	1785	Kónyi gyökér		-	-	-	cattle		„Profert hic comitatus vinum, triticum, sitiginem, avenam, hordeum, pannicum, turcicum triticum, pisa, lentes, millium, vicias, pomorum et fructuum hortensium diversa genera, castaneas, herbam nicotianam, herbam item veneficam Konyi-Gyökér dictam, quae in paludibus Hanság dictis reperitur, Veneficam hujus herbae vim testari possunt tam pecora, quae hujus esu interimuntur, quam et navigantium piscatorum remi, quibus si pernavigantes haec herba transsecetur, venefica ejusdem vis in flammam ex accenso, spiritu vini provenienti similem erumpit."
K. Mátyus 1789	[117]	-	1789	vízi bürök		+	-	-	horse		„a' nyers vízi büröktől mind az emberek, mind a' lovak elvesznek, a' ketskék ártatlanúl észik; az égető ranunculust vagy arannyal-versengő'-füvet a' tehenek el-kerülik , a' juhok örömest megészik, nints-is semmi bajok tőle."
Molnár 1814	[47]	Nagykőrös	1795	vízibürök	8984.4	-	-	-	cattle		„A' vizibürök (*Cicuta* aquatica) ez is felettébb mérges. A' többi két nemei sintsenek méreg nélkül: azért kell vigyázni, hogy az éhes marhákat ollyan kertbe és akolba ne rekeszszék, a' hol sok bürök vagyon, mert külömben nagy kár esketik a' marhákban, a' mint ezt N. Körösön laktamban 1795. esztendőben, egy ittvaló közzbirtokos Titl. Farkas Ferencz Urnak aklában , magam szememmel láttam, és ha oda nem mentem volna, mind a' kilencz vagy tíz tehenei meg döglöttek volna; hármat már a' dögléstől semmiképpen meg nem menthettünk."
Kitaibel in Gombocz 1945	[41]	Ecsedi láp	1796	métely	8100.4	-	-	-	-		" *Cicuta virosa* (ungarisch Métely)"
Földi in Nagy 1976	[29]	Poroszlai tó	1796	tsomorika	8396.1	+	-	-	-		" ezek a vizek kiszáradtak és"bennek a Tsomorika is elveszett … készítenek ebből ma is sokan gyilkos mérget","nem lenne helyes termőhelyeit tilalmi táblákkal megjelölni s ezáltal mérges tulajdonságára a figyelmet felhívni, inkább orvosi hasznáról kell a népet felvilágosítani."
		Téglási rét			8295.4	+	-	-	-		
		Pest határában	1796	*Cicuta virosa*	8581.1	+	-	-	-		„A helytartótanács 1796. szeptember végén közölte a megyékkel, hogy a pesti határban három gyermek *Cicuta virosa*-val jól lakott és meghalt. Az ilyen szerencsétlenségek megelőzése végett elrendelte, hogy a fizikusok működési területükön figyeljék meg, hol terem ilyen vagy más mérges növény és ezekre a helyekre állíttassanak figyelmeztető táblákat"
Linzbauer 1853	[55]	Isaszegh	1796	*Cicuta virosa*	8482.3	+	-	Antidote: milk, fat	-	R	„Noxietas *Cicuta*e virosae at remedia curativa. die 20. Sept. 1796. Ex incidenti repraesenttationis Comitatus Pestensis in eo horsum factae: quod tres pueri in gremiali sua Possessione Isaszegh die 6 Aprilis a. c. ex esu in territorio ejate effossae radicis cicutae virosae, seu aquaticae obierint, quodve ab hoc malo quarta jam adultior, et nominatam radicem haud in tanta, ac tres demortui quantitate sumens puella ope gremialis sui Physici nonnisi per propinatum lac, et pinguia liberata sit, Consilium hoc locumtenentiale regium ad praevertendos similes infaustos casus praett. Dom. Vestris hisce intimandum duxit, ut praeter nota illa generalia vim toxici cujuscunque infringentia oleosa, pinguia, mucilaginosa remedia in similibus casibus in promptu adhiberi solita, insuper ex ratione salutis publicae plagas illas, ubi deleteria ejusmodi venena in majori quantitate crescere solent, per gremiales Physicos suos detegi, et in vulgus notificari faciant, atque erectis etiam columnis, aut tabulis incolas de vitandis idmodi herbis cautos, et securos reddant. Datum Budae ut s."
Kitaibel in Gombocz 1945	[41]	Bag	1796	*Cicuta virosa*	8382.4	-	-	-	-		"Bag - Auch das sehr gefährliche Gift, der Wasserschierling (*Cicuta virosa*) findet sich da ein."
Kiss 1817	[46]	Kónyi tó, Hanság	1797	Kónyi gyökér	8369.4	-	-	-	cattle	R, L	„Mint különös termését a' Hanságnak az úgy nevezett Kónyi gyökeret, méltán lehet itt említeni. A' Kónyi tótól, mivel ottan is terem, neveztetik így. Olly mérges növevény ez, hogy ha a' leveleit a' többi fűvel megtalálja enni a' marha , mindjárt, úgy megdagad tőle , hogy tsaknem felreped a'bőre, 's végtére meg is döglik. A' széllel daraboltatott gyökér a' vízbe vettetvén , meggyúladt pálinka formán futja el a' víznek színét. Ha az ember sebessen kétfelé vágja; akkor is az égerbor' lángjához hasonló lobbanása tűnik szemébe az embernek. Vastag, tsomós mint a' Zeller, ez a' gyökér. Szaga igen nehéz vagyon, szára bürök formájú, vastag, valamint nem is más az, mint faja a' Büröknek (Conium). Elszélledt ágain levelei a' petrezselemhez hasonlók. Hogy elkábuljon vagy el is ájuljon tőle az ember, elég, ha a' kalapjára függeszt egy gallyatskát, vagy levelet belőle."
Kitaibel in Gombocz 1945	[41]	Sarkad	1798	*Cicuta virosa*	9294.3	-	-	-	-		*Cicuta virosa.*
		Pápa	1799	Kony-gyökér	8670.4	-	-	-	cattle		Kony-Gyökér – *Cicuta virosa* – Pápa. – Bihar vármegye"Es wächst hier sehr viel *Cicuta virosa*, die man auch Kony-Gyökér, und in der Biharer Gespannschaft Csomorika nennt; hie starben in wenigen Jahren 12 Ochsen daran."
		Bihar vármegye	1799	csomorika	-	-	-	-	cattle		
Anonymus 1799	[44]	Hanság	1799	Kónyi gyökér		-	-	-	cattle	R	„A' Hanyságbann is valamint a' Kónyi-tó mellett a' Vármegye szélén, bőven találtatik az úgy nevezett Kónyi-gyökér melly olly mérges, hogy ha a' marha meg találja enni, azonnal feldagad 's megdöglik. Hegedűs Úr ezen gyökeret össze aprítva a' Tsatorna vizébe vetette, 's mindjárt úgy el futotta az mérgével a' víz színét, valamint a' meggyúladt égettbort a' láng. Ha az ember sebesen ketté vágja, akkor is az égettbor lángjához hasonló lobbanása tűnik szemébe a tsapáskor. A gyökér vastag, tsomós, mint a zeller, a' levele pedig az igen el terjedt bokros ágainn, hasonlít a petrezselyemhez, mellyet ha le szakasztanak, igen büdös, és ha valaki azt, vagy a szárát tsak kevés ideig is a süvegébenn tartja, vagy hordozza, hamar elkábul s el ájúl."
Kitaibel in Gombocz 1945	[41]	Hídvég	1799	*Cicuta virosa*	9175.4	-	-	-	-		"Hídvég"In der Zala *Cicuta virosa*."
		Homokszentgyörgy	1799	*Cicuta* linearis	9871.4	-	-	-	-		"Homokszentgyörgy: Zwischen dem Wald eine sumpfige Wiese mit einer Menge *Cicuta linearis* nova."
Anonymus 1997	[30]	Szeghalom	1803	csomorika	8993.3	+	-	-	-	R	„Halotti Anyakönyv Ref. Egyház Szeghalom I. kötet. […] 1803. 21. Marty. »Szarka János fia Mihály 6 esztendős. Csomorikát evett és az ölte meg. … A Csomorikát a Házok mellett lévő árok partira találta rajta kívül még egy más Catholicus gyermek is meghót tőle-még kettő evett-de kevesebbett a'kiket hántató által megszabadítottak.«"
Skolka 1997	[52]	Füzesgyarmat	1804	gyilkos csomorika, méregbürök	8893.3	-	-	Antidote: salt	cattle		„Hogy itt nagy mennyiségű vízinövény található, az magától értetődik. Leginkább feltűnő volt számomra a veszélyes gyilkos csomorika vagy méregbürök (*Cicuta virosa* Lin.) nagy mennyisége, amely nemcsak a vízben és a vízparton, hanem a kákával benőtt legelőkön is gyakran tenyészik, és igen nagy kárt tesz a szarvasmarhában. A legnagyobb vigyázat ellenére is elhullik minden évben néhány állat. Az itt lakók nagyon jól ismerik ezt a káros fűfélét, tsomoriká-nák vagy méreg-bürök-nék nevezik (másfelé a *Ranunculus sceleratus*-t nevezik így), és erősen félnek tőle. E méreg kiirtása érdekében, amely ugyan nehéz, de nem lehetetlen, mégsem tettek még semmit. A tapasztalat arra oktat, hogy virágzás előtt mindig sokkal ártalmasabb, mint utána. Útitársam e kiránduláson, Riegler megyei főorvos úr, elkészítette pontosabb leírásukat, mivel az eddigi leírások nekünk nem tűntek kielégítőnek. A botanika barátai számára itt közlöm jellemzését. [...] Ez a leírás minden lényegest tartalmaz: a) Az egész növény kenderhez hasonlatos. b) A gyökér hófehér, egyébként alakja olyan, mintha néhány karórépa nőtt volna egymásra. c) Ha a szétnyomott gyökeret vagy a szétzúzott szárat gyorsan víz alá merítjük, akkor egy különös jelenség jön létre. A víz színén mindjárt egy kéken csillogó felhőcske keletkezik, amelyet az egyszerű emberek lángnak tartanak. Én ezt az állítólagos lángot éterikus olajnak tartom, amely szétdörgölés vagy szétzúzás hatására felszabadul, és a víz felszínén kéken csillogva láthatóvá válik. Ez az olaj oly könnyen elillan a levegőben, hogy a kísérletet nem lehet elvégezni, ha a gyökér csak néhány percig is a szabadban állt. A legjobb ellenszernek (antidotum) a lakosok a sót tartják. Ezért is, ha idejekorán észreveszik, hogy a marhát megmérgezte, erősen dörzsölik a nyelvét sóval, sóval telített vizet is itatnak vele
Diószegi 1813	[23]	-	1813	gyilkos csomorika		+	-	-	cattle	L	„A' gyilkos Tsomorika, minden plánták között leggonoszabb méreg, és kivált a' gyökere, mind az embert, mind a' barmot, kevés idő alatt megöli; mondják mindazáltal, hogy leveleit, a' ketske és a' juh, ártalom nélkül megeheti. Terem a' lápokbann és posványokbann, és tsak azért jo esmerni, hogy a' hol az ember találja, irtsa ki."
Dóczy 1830	[109]	Hanság	1813	Kónyi gyökér		-	-	-	-		„A' sok le tsapolásokat, és tsatornákat, mellyek a' Hanság vizeit a' Rábtzába vitték; az 1813 esztendei ki áradások haszontalanná tették. Említtést érdemel itt még az úgy nevezett igen mérges, és veszedelmes kónyi gyökér, (*Cicuta virosa*) melly tulajdon képpen egy majd fára nevelkedő vízi maszlag."

Source	Ref. (1)	Location	Date	*Name*	Code (2)	Human pois. (3)	Misid. (4)	Ethnom.(5)	Anim. (6)	Plant part (7)	Original text
Récsi 1854	[142]	-	1813	*Cicuta virosa*		-	-	medicinal herb	-		„Egy 1813 oct 2 ról kelt alsó ausztriai kormányszéki rendelet Prov GS f Nieder Oesterr 1829 2 rész 771 lap által közzétetetett azon növényeknek jegyzéke melyeket a gyógyfüveseknek csak különös ovatosságok megtartása alatt szabad eladniok s melyeknek közönséges kézi eladása tiltatik Ezek Capsulae papav somnif Solanum nigrum Caules Dulcamarae Datura Stramonium Hyosciamus niger Hyosciamus albus Lolium temulentum Ervum Ersilia Chenopodium hybridum Lactuca virosa Prunus Laurocerasus Paris quadrifolia Atropa Belladonna Digitalis purpurea Chaerophyllum silvestre és temulentum Aethusa Cynapium Sium latifolium és angustifolium *Cicuta virosa* Conium mac és ersaticum Ledum palustre Mercurialis perennis Bryonia alba és dioica Colchicum autumnale Plumbago europaea Cynanchum erectum Cyclamen europaeum Hydrocotyle vulg Oenanthe fist és crocata Alisma plantago Clematis flamula erecta és integrifolia Aristolochia clematis Anemone pulsatilla pratensis és nemorosa Helleborus niger viridis és foetidus Veratrum album Caltha palustris Aconitumnak minden fajai Daphne Mezereum thymeola és laureola Arum macul Euphorbianak minden fajai Ranunculusnak minden fajai Raphanus Raphanistrum Gratiola Asarum europaeum Cortex interior et turiones Sambuci Sabina Phellandrium aquaticum Veratrum nigrum Arnica tana Chelidonium majus Rhus radicans és toxycodendron Semina Ricini Scilla marina Secale cornutum”
Ángyán 1816	[31]	-	1816	csomorika		+	-	-	-		„Az Isteni — gondviselésnek , a világon levő gonoszokból vett ellenvetés ellen való megigazitása. — Egy merges Plánta’ - nevezetesen a Tsomorika étele miatt - megholt gyermek felett.”
Magda 1819	[124]	Békés vármegye	1819	csomorika, méregbürök		-	-	-	cattle		„Természeti állapotja. Békés Vármegyében tsupa térségnél egyebet látni nem lehet; hegynek hire sints. […] Azon mérges plánta, melly itt tsomorikának vagy méreg büröknek (*Cicuta virosa*, Wasserschierling) neveztetik, sok marhát meg mérgesít.”
Wierzbicki in Bodnár 1957	[99]	Fenékpuszta	1820-1826	*Cicuta virosa*	9269.3	-	-	-	-		„ *Cicuta virosa* Fenékp.”
Bertalan Szilágyi 1920	[53]	Nagy-Sárrét, Nagybajom	1827	gyilkos csomorika	8893.1	-	-	Antidote: salt, urine	sheep	R	„Mindazáltal megjegyzésre méltó 1-ször a gyilkos csomorika (*Cicuta virosa*), mely bőven terem ezen rétségnek mellyékén, még pedig ott, ahol a szarvasmarhák is hasig érő vizben a sások között legelnek és ezen plántának leveleit is minden ártalom nélkül lerágják. Torzsájában van csak a megölő méreg, mely ollyan formájú, mint a karalábé. Ennek próbatételében magam egy virtsének gyilkosa lettem. Három s négy kis szeletet belőle szájába beadván, mellyeket jó izűn bé is falatozott, de alig tölt el öt minutum, reszketésbe jött ártatlan foglyom és nagy rángatódással terült el a földön. Szántam szegényt, de kívánságomnak néki kellett áldozatja lenni. A csomorikának mérges torzsáját, mely édes ízűn ízlel, csak a juhok szeretik csemegélni, de ez is csak téli időben szokott megtörténni, mikor a rét fagyban lévén, a juhászok nyájaikat a rétbe is bebocsájtják és ha valamely juhot észrevesznek, hogy csomorikát evett és ledobbant, a már nállok készen lévő orvoslását, a sót szájába benyomják, vagy pedig, ha az nincs, szájába pesel és semmi baja nincs.”
Kiss 1961	[122]	Kék	1827	mételytorzsa	7897.3	-	-	-	cattle		„Kék 1922. évi gyűjtés […] Az 1827 conscriptio szerint 7 éves átlagos számítás szerint 10 marha pusztul el mételytorzsától.”
		Vasmegyer			7896.4	-	-	-	cattle		„Vasmegyer 1923. évi gyűjtés […] Jószágkiálló kiálló helyekül ismerték Szilasszeg, Jakabsziget, Hosszúsziget, Pecertéssziget és Kisapáti helyeket. A tavak szélein mételytorzsát kapott a jószág, a homokhegyek közötti mélyedésekben, gödrökben, a fodorsásban pedig pókot, amitől elpusztult. Az 1827-iki összeírásban ezt olvashatjuk: »A Tisza áradásainak és határ területének nagy része minden évben ki van téve. A kiöntések által úgy a földesúri, mint a jobbágyterület nagy része víz alá jut, azon kívül hét éves átlag számítás szerint minden évben két marha szokott elhullani a dögletes füvek, különösen a mételytorzsa élvezete miatt, amely a legelőkön nő.«”
Fazekas 1994	[112]	Ökörítő	1828	mételytorzsa	8001.3	-	-	-	cattle		„Ökörítő jobbágyai szerint "a marhák mintegy egyharmad része minden 4-dik esztendőben dögben elvesznek, melyet részben a legelőkön lévő számtalan pókok sokasága, részben a mételytorzsa okoz, amely olyan veszedelmes plánta, hogy a vízben is meggyül, melyet a marhák igen szeretnek, a lápban pedig bőven szokott teremni"
		Győrtelek, Tyukod			8000.4	-	-	-	cattle		"Ura, Tyúkod, Györtelek, de a Szamos menti községek vallomásaiban is elhangzott, hogy a mételytorzsától az állatok felfúvódnak, vagy ahogy a tyukodiak fogalmaztak "dögletes víz szaporodott össze a marha bőre és húsa között", és hamarosan tömegesen elpusztultak.”
		Ura			8101.3	-	-	-	cattle		
Anonymus 1828a	[32]	-	1828	gyilkos csomorika		+	-	-	-	S	„De vannak köztök némellyel, mint a’ gyilkos Tsomorika (*Cicuta virosa* L.), és a’ büdös Bürök (Conium maculatum L.), mellyek minden részeikben mérgesek. Hányszor nem tsinálnak belőle sipot a’ gyermekek , ’s így hányszor nem veszik szájokba azon veszedelmes mérget, melly ha az életet egészen meg nem töri is, de bizonyosan az egésségnek , kivált az agyvelő - rendszernek kárára vágynak!”
Csaplovics 1829	[107]	Hanság	1829	Konyer Wurzel		+	-	-	cattle	R, L	„Länge gezogenen Neben Canäle Bemerkenswerth ist eine Giftpfianze welche im Hanság wie auch in den Sümpfen Niederungerns häufig angetroffen wird Sie heißt Kónyi Gyökér Kónyers Wurzel vom Kónyer Teiche wo sie häufig wächst Diese Pflanze ist sehr giftig und ihre Wirkung bei Menschen und Thieren schnell tödtend Das Vieh welches nur wenige Blätter davon mit dem Grase frißt schwillt schnell bis zum Zerplaßen auf und stirbt Man darf nur ein Blatt oder ein Stück von der Wurzel auf den Hut heften so wird man davon bis zur Ohnmacht betäubt Wirft man die Wur zel zerbröckelt in's Wasser so überläuft den Wasserspiegel ein Schein ähnlich der Flamme des brennenden Brannt weins und wenn man die Wurzel schnell entzwei hackt zeigt sich derselbe Schein welcher bald verschwindet Diese Wurzel ist nichts anders als der große Wasserschiers ling *Cicuta virosa* dessen Wachsthum hier außerordents lich üppig ist indem er Baumhöhe erreicht und sein Gift nimmt im Verhältniß seiner Größe zu Die Wurzel ist sehr groß dick und knotig inwendig weiß voll kleiner Höh lungen mit weißlichem Saft angefüllt von außen hat sie Ringe aus denen Haare hervorbrechen welche im Wasser schwimmen oder im Schlamme wurzeln die Pflanze ist hoch und stämmig Blätter und Saamen gleichen denen der Pe terfilie Der Geruch ist ekelhaft und betäubend.”
Polgár 1941	[139]	Ravazdi tó	1830	kónyi gyökér	8472.4	-	-	-	-		„ *Cicuta virosa* L. – olim; Csapó 51”Győr vármegyében egy helyen bőven terem”, Kóny (J. J. Torkos ap. Haberle Succinta rei herb. 1830 p. 16), J. Leop. von Stocz, Das Königr. Ungarn, Wien 1823 p. 164 et 176”Kónyi gyökér”; Ravazdi-tó (nunc non existit) Ball. ap. Eb. 112). – In praesentia haec species in territorio nostro deficere videtur.” (= manapság ez a faj a mi területünkön eltűnni látszik)
		Kónyi tó (Hanság)	1830	kónyi gyökér	8369.4	-	-	-	-		
Szotyori 1831	[146]	-	1830	vízi mérgesbürök		-	-	-	cattle		„Vízi mérgesbürök (*Cicuta virosa*) , nem annyira hasznairól , mint ártalmairól ismeretes ; árt embernek és marhának ; jó vólna kiírtatni a ' pásztori gyermekekkel”
		-	1830	vízi mérgesbürök		+	-	-	-		
Kassai 1834	[118]	Szerencs	1833	mételv	7893.3	-	-	-	-		"mételv: mérges vízi bürök, gyilkos csomorika (*Cicuta virosa*) "
		Hegyalja	1833	mételfű	7794.4	-	-	-	-		„Mételv fü a Hegyaljánn *Cicuta* Virosa”
Béres 1976	[98]	Hortobágy	1834	csomorika		+	-	-	cattle		„Hortobágyon különösen nagy gondot okozott a vízhiány. Egyik másik gulya a víznek nem léte, a szomjúság miatt teljesen leromlott.” A dögletes veszély több gazda véleménye szerint a”legelőn található mérges plántáknak, nevezetesen Csomorikának tulajdonítódhatik”, amely az emberi egészségre is ártalmas lehet, ezért a mészárosok csak az orvosok által megvizsgált állatokat vághatják le.”
Anonymus 1834	[92]	-	1834	gyilkos csomorika		+	C	-	cattle	R, L	„Gyilkos csomorika (cicuta virosa) […] Ezen füvet táplálékkal felcserélni nem könnyen lehet, a’ különböző terméshely miatt. Gyöke néha igen nagy, melly többnyire alakjában zellérgyökérhez, szagában peszternákhoz, izében petrezselyem gyökérhez hasonlít, ’s ezen gyászos hasonlat miatt még is sok szerencsétlenség történt. Nem régiben is zellernek nézvén egy szegény asszony, megfőzte és két gyermekével evett belőle. A’ kisebbik meghalt, a’ nagyobbik csak nehezen szabadittathatott meg a’ haláltól, maga pedig az asszony sokáig súlyosan betegeskedett. Belől fejéres húsa van ezen növény gyökérnek, és tele üreges hézakokkal, mellyekből ketté vágáskor sáfrányszinü nedvesség foly, eleinte egy kissé undorító ízzel, utóbb pedig a’ nyelven égető csípősséget okoz. Télben és tavasszal legmérgesebb ez a’ nedv, ’s ha egy éjszaka áll, egészen megveresedik. Ezen nedv legerősebb mérge a’ növénynek; még az állóvízzel is közösül, hol bőven terem,s annyira megrontja azt, hogy gyakran oka lett kimagyarázhatatlan marhadögnek.”
Anonymus 1835	[93]	-	1835	*Cicuta virosa*		-	-	-	horse		„Emlitést tészek a növények országából a többek között … a gyilkos csomorika (*Cicuta virosa*).”
Anonymus 1836	[56]	Hanság	1836	Kónyi gyökér		-	-	-	-		„Több helyein a’ Hanságnak egy igen mérges gyökér is találtatik, ’s kónyi gyökérnek — *Cicuta virosa* — neveztetik, minthogy legnagyobb mennyiségben a’ kónyi tó körül terem. Kevés adagban beadva is halálos következményű. Párna alá tétetvén , a’ rajta fekvő ember elájul; sőt még közelítni sem tanácsos hozzá, mert szaga is kedvetlenül hat az emberre.”
Anonymus 1838	[36]	-	1838	csomorika		+	-	-	-		„Erről mondja a’ népdal: Ki az urát nem szereti, Csomorikát főzzön neki, Ha ma vele megeteti, Még estvére kiteríti.”
Fényes 1839	[45]	Békés vármegye	1839	csomorika (méregbürök)		-	-	-	cattle		„Egyébiránt a bőven találtató csomorika (méreg-bürök) veszedelmes a szarvasmarháknak.”
Joachim 1841	[119]	-	1841	csomorika		-	-	against TCL (as anaesthetic)	-		„Gyakran czélszerü e gyógyszert, az idegek érzékenységét tekintetbe vévén, — bódítókkal összekötni. Mellyik választassék ezekből: az egyediség, testalkat, és kórtünemények határozandják meg. — Sok esetben a mérges csomorika (*Cicuta virosa*) kitűnő szolgálatot tesz; mi több, e szert oldozó erejeért némelly orvosok különhatásosnak hirlelik a görvélyben.”
Papp 1841	[132]	-	1841	csomorika		-	-	-	cattle		„Sőt tegyük fel, hol a vérvizellést olly növényektől kapják marháink, mellyeket a természet kebele nem a szarvasmarhák részére tart, azokat meg nem eszik s ha megeszik azoktól bántatnak s talán halálosan is, az illy növényeket természeti ösztönénél fogva megösmeri ugyan a marha s ennél fogva meg nem eszi, de tegyük fel, hogy a szabadban véletlenül falatja közé keveredik egyik vagy másik azok közül mellyeket nem tűr p. o. élesmosó és szürke fenyér (Andropagon gryllus et ischaemum) lófarkú uszszakál (Hippuris vulgaris), csíkos kecskerágó (Evonymus europaeus), maszlagos haramag (Oenanthe crocata), gyilkos csomorika (Cicuta virosa) zsurló (Equisetum) s vérvizellést kap.”
Kubinyi 1842	[19]	Veres-egyháza	19th c.	gyilkos csomorika, vízibürök	8381.4	+	Pp	-	-	R	"Két fiú és hat leány a gyilkos csomorika gyökerét, mellyet a sertések a földből kitúrtak, kerti pasztinák (Pastinaca sativa) helyett megették. E szerencsétlenek közül csak hamar többen iszonyú halállal múltak ki, és csupán azok maradtak nagy nehezen életben, kik jókor hánytak. "
		Keresztúr	19th c.	gyilkos csomorika, vízibürök	8581.1	-	-	-	cattle		"Gyakran tapasztaltatott, hogy a lovat, szarvasmarhát és juhot is megöli, ha talán vigyázatlanságból nekie eledelül nyújtatott. Hol e növény nagy mennyiségben terem, mint Magyarország sík részének mocsáros helyein, nevezetesen Bihar megyében, Pest megyei Keresztúr s Veresegyház körül, ott a víz fölött valamelly olajhoz hasonló folyadék lebeg, mit a gyökér bocsát ki. Elmérgesedvén ettől a víz, ez mind az embernek, mind pedig a marhának veszedelmes lehet. S ugyanezért némelly tartományokban kiirtása keményen parancsoltatik."
Téglási Ercsei 1842	[148]	-	1842	gyilkos csomorika, vízi bürök		+	P	-	cattle	L	„Egy falus bírónak három leánya vacsorára levest főzvén peterzselyem levelei helyett ezen bürök leveleit apritják a fazékba Annyuk mind a hármat reggel merevedve találja Szerencsére érkezik egy orvos ki kettőt közülük életre hoz de a harmadikat a haláltól meg nem mentheté. Hozhatnánk több rettenetes példákat is elő, de ennyi legyen elég figyelmeztetésül. Szarvas marhák és lovak kerülik ugyan a vízi bürköt, de ha szorultságból megeszik marha dög lesz következése”
Anonymus 1901	[94]	Bihar vármegye	-	*Cicuta virosa*	-	-	-	-	cattle	-	„A legelő füvében sok *Cicuta virosa* volt, a mely kora tavaszszal még igen apró s valószínűleg hiányzik az a specifikus szaga, a melyet az állatok felismerhettek volna. A tehenek egy része néhány óra múlva elhullott, a többi két nap alatt visszanyerte egészségét. A tulajdonosok e tehenek tejét minden hátrány nélkül fogyasztották.”
Peller 1843	[136]	Bihar vármegye	-	gyilkos csomorika, méregbürök	-	-	-	-	-	-	"Csomorika *Cicuta* L … Gyilkos Cs. (*C. virrosa* L.) Gyökere répanemű belül hézagos. szára tövön vastag. … gyökében sárga nedv foglaltatik (méregbürök). Orv. haszn. füve. Gyógyereje: kis adagban enyhitő oszlató, nagy adagban széditő, kábító mérgező."

Source	Ref. (1)	Location	Date	Name	Code (2)	Human pois. (3)	Misid. (4)	Ethnom.(5)	Anim. (6)	Plant part (7)	Original text
Máttyus Nepomuk 1845	[126]	-	1845	csomorika, vízibürök		-	-	-	horse		"Mérges növények […] Csomorika (gyilkos), vízibürök, Wasser Schierling "
Boross 1857	[104]	-	1857	csomorika, vízibürök, mérges ádáz, vízi bősövény, halálgyökér		-	C, Pp	its squeezed juice is an effective remedy for glandular nodules, runny boils, skin rashes	-		„Vizibürök vagy gyilkos csomorika, méregbürök, vizi bősövény, mérges ádáz, halálgyökér. Ezen huzamos életű növény álló és folyóvizekben mocsárokban posványos árkokban, tavak iszapos partjain, folyók és patakok mellett szokott tenyészni. Juliustól augusztus végeig virágzik. Ennek megismerésénél minden ismertetőjelre szorosan kell ügyelni, mert tavaszszal zeller és őszszel petrezselyemgyökérhez hasonlít szaga hasonló a pasztinákéhoz peszternák s olyanforma íze van, mint a zellernek. Tudatlan s ismeretlen ember leginkább ezzel zavarja össze s e helyett eszi meg. Gyökere nagy bütykös, körül domború gyűrűk környezik s folyó gyökszirmokkal boritott minél öregebb lesz annál jobban elterül, belül üreges csipős sárgás tejnedvvel gazdag, mely a gyökér fölvágatásakor a levegőn vörösessé válik. És ezáltal lehet legjobban megkülönböz tetni a zellertől, mert annak nincs ez a tulajdonsága. Szára gömbölyű csőalakú, vastag, két s négy lábnyi magas. Levelei lándzsa alakuak, fűrész módra csipkézettek fényes sötétzöldek és a levélszárak hüvelyformára körítik az ágakat. Virágai épen a levelek fölött egyenesen összeillesztett fehér ernyőt képeznek. Kelyhe ötfogú, koronája fehér, öt megforditott vese és szivalaku levelekből alkotott. Gyümölcsének nincs hüvelye, gömbölyü magva tojásdad, színre s izre hasonló a petrezselyemhez. Gyökere és szára legmérgesebb. Bevétele nyomó és égető gyomorrágást, borzasztó görcsöket, őrületet, szótlanságot, rángatózást s hirtelen halált szokott okozni. Gyökerének és szárának tejszerű nedve még azon vizet is megmérgezi, melyben az növény terem. Ellenben ezen növénynek kifacsart nedve hatalmas orvosság mirigy csomósodásoknál, folyó keléseknél s bőrkiütéseknél.”
Kátai 1858	[51]	-	1858	vízibürök, gyilkos csomorika		+	P	-	-	R	„A vizi bürök vagy gyilkos csomorika (*Cicuta virosa*, L. Wasser-Schierling), minden hazai mérges növényeink legmérgesebbike, mocsárokban, árkokban s folyók partjain terem. Legnagyobb ereje vastag, belül üres, vagyis hézagos izekre osztott fehér gyökerében rejlik, melly ha megmetszetik, tejszinü, majd halvány sárga, később sáfrány szinűbe átmenő, égető izü, orrtekerő nedvet bocsát magából. Azon szerencsétleneket, kik ezen gyökeret vagy tudatlanság- vagy vigyázatlanságból a petres nyem igen hasonló gyökerével felcserélve megeszik — … a leggyorsabb idő alatt s iszonyú kinok közt szokta kivégezni, mert a gyomorba vitetve iszonyú hőséget, fájdalmakat s kielégithetlen szomjúságot, gyomorgyuladást, csuklást, görcsöket, szédülést, ittassághoz hasonló szédelgést, rángásokat s végre halált okoz. Halál után a has iszonyú nagyságára felpuffad, a gyuladásba jött bélrészek és gyomor, fenésedésbe mennek át, s ez okból összerágatnak, az agy edényei vértől telülve duzzadtak.”
Melkay in Nebojszki 2017	[129]	Bácsalmás	1860	gyilkos csomorika	9882.3	-	-	-	-		„A mocsarakban vagy mellette nőnek […] *Cicuta virosa* Gyilkos csomorika […] Melkay György, a település jegyzője. Almás 1860.”
Argenti 1863	[95]	-	1863	*Cicuta virosa*		-	-	against concussion, seizures, feeling of being cramped, convulsive lethargy, nervous tension	-		„Az agyrázódás, agymozzanás kül erőszak hatása után azonnal szédülést, tompaságot, lelki tehetségek elenyésztét, vagy azonnali halált okoz. Hatalmas szer az agyrázódásnál az arnica, melyből öt gólyát tíz evőkanályi vízben felolvasztván, eleinte adhatunk minden V órában egy kanállal, javulást érezvén, minden 1. 3. 5. órában. Hasznos itt a *Cicuta virosa*, ha az arnica tán nem lenne elegendő, szintén úgy veendő.”
Nagy 2001	[38]	Hajdú-szoboszló	1868	gyilkos csomorika	8594.3	+	-	against TCL	-	R	„Özv. Agárdi Jakabné 54 éves szoboszlói tojás- és csirkekofa beismerte, hogy 1868. június 13-án Maga János 6 éves leánykáját - szülei kérésére gyógyítás alá vette. A gyermek sorvadásban és az orvosi vélemény szerint nagyfokú, életéhez kevés reményt adó görvelykórban (a nyaki nyirokmirigyek megduzzadásával járó kór) szenvedett. A vádlott gyilkos csomorika gyökeréből tejben főzetet készített, abból bizonyos mennyiséget beadott a gyermeknek, aki ennek következtében aznap este nagy kínok között meghalt. A szakképzetlenek gyógyító tevékenységét a fennálló szabályok szigorúan tilalmazzák, - állapította meg a törvényszék. Vádlott tehát törvényellenes gyakorlata közben emberölésben vált bűnössé. Tekintettel arra, hogy tilos kuruzslásait már korábban is űzte s a gyökérrel, amely különben a senyvedt s alig életképes gyermek halálát előidézte, a legmérgesebb növények közé tartozik, ezért 6 hónapi rabságra ítélte.”
Frank 1870	[113]	Tata	1870	*Cicuta virosa*	8376.3	-	-	-	-		„míg tovább az igen laza homokos tóparton, s a mellette fekvő földeken találtatik: […] *Cicuta virosa* L.”
Pokorny & Dékány 1873	[137]	-	1873	gyilkos csomorika, Wasserschierling	-	+	Ca	-	-	R	„A gyilkos Csomorika … *Cicuta virosa* … Ezen növénynek a répához hasonló gyökerével történik leggyakrabban a megmérgezés jóllehet haránt rekeszezett gyökerét nagyon könnyen meg lehet különböztetni minden élvezhető gyökértől mérges növénytől. … elég gyakran előfordul ugyan a tavak és folyók partjain de szerencsére nem mindenütt.”
Soltész 1873	[28]	-	1873	vizibürök, mérges csomorika		+	-	-	-	R	„A mérges csomorika (*Cicuta virosa*), … Vízárkok mellett, tókban, posványokban, áradásos helyeken találtatik, s innen vette vizibürök nevét. Egyike a legerősebb és veszélyesebb belföldi méregnövényeinknek, melynek kissé fűszeres, de bódító szagú gyökerével már igen sok mérgezés történt főkép gyermekeknél, kik azt más megehető gyökökkel cserélvén föl, vigyázatlanságukért többnyire halállal lakoltak. De egyéb része is mérges, sőt puszta gőzölgésével is képes szédülést okozni.”
Ebenhöch 1874	[111]	Ravazdi tó	1874	mérges csomorika	8472.4	-	-	-	-		„*Cicuta virosa* L. (Mérges Csomorika.) Ravazdi tó.”
Széchenyi 1876	[145]	Fertő-tó	1876	Kónyi gyökér	8366.2	-	C	-	cattle	L	„Létezett még valaha egy csodálatos mérges növény is, az u. n. kónyi gyökér (Konyer Wurzel), mely a bódító maszlagos nadragulya osztályához tartozott. Hasonlított a zellerhez, gyökerei vastagok, gümősek és erős szagúak voltak, széthasított levélszárai egészen a bürökhöz, végük pedig a petrezselyemhez hasonlított. Ha a barom evett belőle, azonnal felpuffadt és elveszett. Hogy az ember elbóduljon és elájuljon tőle, elég volt egy levelét megszagolni, vagy a kalap mellé tűzni.”
Chernel 1877	[106]	Kőszeg	1877	gyilkos csomorika	8565.3	-	-	-	-		„ *Cicuta virosa*. Gyilkos csomorika.”
Dubay 1878	[20]	-	1878	*Cicuta virosa*		+	C	-	-	R	„Oda érkezve a szakácsnét a konyhában félig elalélt állapotban, kábultan elterülve találtam, az émelygésekkel és hányásingerrel küzdve; a lakszobában ugyanily állapotban P. F-et és nejét, 14 hónapos kis fiók pedig sötét piros duzzadt arccal látszólag nyugodtan aludt a bölcsőben. […] Krumpli, kalaráb, sárgarépa és petrezselymen kívül két meglehetős nagy gyökér vonta magára figyelmemet, melyek a zelleréhez külsőleg igen hasonlítottak, de megszagolva a büröknek sajátságos szagát árasztották és hosszában vágva nem tömött sárgás belet — mint a zellernél — hanem fehér hoszúkás sejtü állományt találtam, mely ama sajátos szagot, kellemetlen kesernyés s kissé csípős izt még inkább jelezte. […] A levesmaradéknak közelebbi megvizsgálása kimutatta, hogy ama két gyökér a *Cicuta virosa* gyökere volt és zeller helyett került a levesbe egy zöldséget áruló asszonytól”
H. Arányi 1880	[26]	-	1880	gyilkos csomorika, vízi petrezselyem		+	P	-	-		„Vizi petrezselyem vagy gyilkos csomorika (*Cicuta virosa*) ezt is főzték már petrezselyem helyett levesbe, és volt eset reá, hogy az egész család kihalt utána.”
Borbás 1881	[100]	Szarkaszige t	1881	*Cicuta virosa*	9093.2	-	-	-	-		„Kót és Iráz […] *Cicuta*” […]”A nádasok szélében boros (süppedő sáros) terek is maradnak, ahol a phanerogam vegetatio buja: óriás *Cicuta virosa*, Sium latifolium, Oenanthe aquatica, Symphytum uliginosum, Butomus umbellatus (Iráz).” […]”Szarkaszigeten Szgh. 1881!”
Czirbusz 1882	[108]	-	1882	csomorika		-	-	medicinal herb	-		"A betegségeket szintén ráolvasás. Sympathikus szerek (a betegségek másokra, p. fákra, ruhadarabokra átvitele) és gyógyfüvekkel gyógyítják, ha azok sem használnak, misét olvastatnak a beteg felüdüléséért. A gyógyfüvek között nagy szerepet játszik a csomorika (*Cicuta virosa*), a hagyma, paprika, bodzavirág, a Chamomilla, a Berberis bogyói, továbbá az Absynthium pálinkában vagy vörös borban.”
Oláh 1888	[33]	Sarkad	1888	csomorika fű	9294.3	+	-	-	-		„Hallotta, igy szólt – a Kovács István szolgájától, hogy asszonya Kovács Istvánné adott egy máriást Gréczi Jánosnénak, hogy hozzon neki csomorika nevű füvet, ezen fű felől pedig azt hallotta, hogy a mely embert megfürösztenek benne, az a halált el nem kerülhetné. Azt pedig tudja hallomásból, hogy Kovács Istvánt azon fűből csinált fürdőben mosták meg, de hogy ezen dolgot valaki tanácsul adta volna Kovács Istvánnénak, nem tudja. […] »Hallottam, úgy szólt, Gréczi Jánostól nyilván, hogy két véka búzával fogadta meg ötét Kovács Istvánné, hogy Sarkadra menne csomorika fűnek hozásáért. Gréczi János pedig szegénységéhez képest elmenvén és el is hozván, tudta azon fűnek ártalmas hatalmát, mondotta Kovács Istvánnénak : »Meg ne förözd az uradat azon fűben, mert mindjárt meghal.«”
Deininger in Mágócsy-Dietz 1889	[110]	Lébény	1889	csomorika	8269.4	-	-	-	-		"A Hanság növényzetét illetőleg engem is érdekelt a felvetett kérdés, amennyiben a mosonmegyei részen is azt tartották egy időben, hogy az ottani mocsári flóra, legeltetve, mérges hatású a marhára; egy ízben a lébenyi szarvasmarha-falkát derékig érő vízben követtem, meglesendő, mely növényekkel táplálkozik főleg, de nem tudtam egyetlen esetet is megfigyelni, hogy az ott nagy mértékben diszlő *Cicuta* virosából (csomorika) harapna ; a marha azt ösztönszerűleg mellőzi , hanem igen is evett nádhegyeket elsősorban, másodsorban pedig az ott zsombékokban előforduló sásokból különösen az ott gyakori Carex stricta, C. paludosa és C. acuta fajokat ; de mindezen növények nem mérgesek."
Pap 1893	[131]	Szeged	1893	csomorika	9787.1	-	-	-	-		„A nádasok és a mocsárok tenyészetének kiegészítői … Csomorika.”
Horváth 1895	[48]	Tarcal	1895	sás, mételytorzsa	7994.1	-	-	-	cattle	R, L	„Legyenek szívesek e csomagban foglalt, Tarczal környékén sásnak nevezett gyökgumót behatóan megvizsgálni, s nevét velem tudatni. Gazdatisztek e növénygyökeret mételytorzsának hívják itten. Alkalmatlankodni pedig azért bátorkodom, mivel különösen furcsa sajátságát tapasztaltam e növénynek. Ugyanis határunkban sok és nagy tó van, melyek partján igen nagy mennyiségben fordul elő e növény. Mikor a tóvíz őszkor leapadt, a szarvasmarha hozzáférhetett, s meglehetős mohón ette; csakhogy ha jóllakott vele, rövid idő elteltével elhalt, így egy uradalomban két tinó dobkórra emlékeztető tünetek közt múlt ki mintegy 5—6 óra elteltével, melyeket az uradalmi tiszttartó felbonczoltatott. E bonczolásnál következőket tapasztaltam: A leendő gázok miatt rendkívülien fel volt fúvódva, a százrétű igen szárazon s keményen tömve volt sáslevelekkel s a nevezett növény gyökérrészeivel is. A tüdő vérrel s kevés levegővel volt telve. A rekeszizom mindkét esetben szorosan a tüdőig volt nyomulva. A többi szerv ép volt. A beendő, bonczolás közben megszúratva, trombitaszerű harsogással gázt lövell ki, épen úgy mint a dobkórnál. Vájjon milyen hatóanyag van ebben a növényben? Vájjon az ecclampsia ezen növény által keletkezik-e, mert lóherétől vagy luczernától származó felfúvódásnál ily tüneteket nem láttam, nevezetesen hogy az állat mintegy szédülve körbe forgolódik, azután földhöz vágódva egyet bömböl, szeme felfordul s rövid idő alatt kimúlik.” [...]”A beküldött növényrész semmi egyéb mint a gyilkos csomorika (*Cicuta virosa* L.) gyöktörzse (tőkéje, rhizomája), melyet a belsejében egymás felett levő üregekről könnyű megismerni, ha hosszában átmetszük. A marha gyomrából kiszedett levelek közt volt sáslevél is, de több csomorikalevél is.”
Takáts 1899	[147]	Ecsedi láp	1899	mételytorzsa	8100.2	-	-	-	-		„A vizek legelterjedtebb növénye a nád volt s amig valami, nálánál erősebb hatalom ki nem szorította helyéből, más növényt nem tűrt meg maga mellett. Csak a sas tudta bevenni s elfoglalni birodalmát. Amint a viz magasabb lőn s elbobta a nádat, a kalákány vette át az uralmat. Óriási területeket borított el s oly sűrű volt, hogy hajóval sem lehetett keresztültörni rajta. Imitt-amott a lómenta, a búzalevelű fa, a mételytorzsaa, nadálytő, futófű, lósóska, papvirág, kangyékény, zsom, aztán a taraczkos-, pallagi- és sudársás megvegyült vele s még sűrűbbé lett a viz színén úszó növényréteg.”
Virágh 1899	[149]	Hanság	1899	Kónyi gyökér		+	-	-	cattle		„Ámde ha e lápvidék sok jóillatú növénnyel kedveskedett, úgy olyannak se volt híján, mely nem hogy gyógyított volna, mint sok itteni növény, hanem megölte az embert. A Kónyi Gyökér mely nevét a »Kónyi Tótul« vette, legveszélyesebb növénye volt a Hanságnak. Ott azonben nem »Conium«-ról van szó, hanem a » *Cicuta virosa*« a bürök legdühösebb fajáról, melytől a marha is elpusztult.”

Source	Ref. (1)	Location	Date	Name	Code (2)	Human pois. (3)	Misid. (4)	Ethnom.(5)	Anim. (6)	Plant part (7)	Original text
Borovszky 1901	[105]	Bihar vármegye	1901	*Cicuta virosa*	-	-	-	-	-		„A növények a vízben gyökereznek, de különben egész termetükkel a vízből kiemelkednek, gyakran a parton s közelében is vegetálnak: […] Cicuta virosa"
Borbás 1901	[101]	Szarkaszigeт	1901	*Cicuta virosa*	9093.2	-	-	-	-		„Az úszó sziget keletkezésére és megvizsgálására hazánkban ma ritka az alkalom, mert számos vizet lecsapoltak s vele a fűéletnek számos tanulságos jelenségét megsemmisítették. A letűnt század hetvenes éveiben még a Sebes-Körös Sárrétjéről, Békés és Bihar vármegye határáról emlegették, de már én 1877. óta a nyomába jutni nem tudtam. Sok kérdezősködés után Szeghalom határában a Szarkaszigethez utasítottak, ott lehetett volna még látni az úszó szigetet, ahová állítólag a víz kivetette. Itt a nádas szélén valóban egész rakás nádhulladékot láttam, mely a téli nádvágásból torlaszolódott össze s egy ideig a Kőrös magával sodorván, itt a partra vetette. A nádtöredéken és körülötte *Cicuta virosa*, Sium latifolium, gyékény, Bidens cernuus, keserűfüvek, Lycopus Europaeus, vízi lósóska stb. buján nőttek, de sem itt, sem a közel süppedősebb helyen semmi jellemző füvet nem lehetett látni."
Wagner 1902	[150]	-	1902	gyilkos csomorika, mérges bürök, vízi bürök, métely torzsa					cattle		„Gyilkos csomorika. — Cicuta virosa L. — (Mérges v. vízi bürök, métely torzsa.) —- 157. kép. 2\. 125 cm. Húsos tőkéje ferde és harántfalakkal üregekre osztott. Levelei háromszorosan szárnyasak, élesen fűrészesek, keskeny, szálas-lándsás levélkékkel. Gallérja nincs, gallérkája soklevelű. Ernyője 10—15-ágú; pártája fehér ; ikertermése gömbölyded. Terem szórványosan nagyobb és állandó mocsarakban, vizenyős réteken. 6—8. Cicutin - tartalmánál fogva hazánk legmérgesebb növényeinek egyike. Irtását Németországban a törvény parancsolja ; nálunk aránylag ritka és csak helyenként fordul elő nagyobb mennyiségben. A legelő marhára is mérgező hatású."
Polgár 1902	[138]	Győr	1902	*Cicuta virosa*	8371.4	-	-	-	-		„Ritkábbak a *Ranunculus lingua, Cicuta virosa,* stb. Ezen növények közt kevés van, melyet Közép-Európa bármely nádasában nem lehetne ép úgy megtalálni, mint vidékünkön."
Szabó 2002	[144]	Tapolcza lápteknő	1905	mérges csomorika	9170.2	-	-	-	-		„*Cicuta virosa* - Mérges csomorika: Tapolcza lápteknő 1905 július 20, … Mocsarakban, fűzlápokban."
Gombocz 1906	[114]	Kecske-patak	1906	*Cicuta virosa*	8365.1	-	-	-	-		„*Cicuta virosa* L. Mocsarakban, patakok mellett Sopron m. a Kecskepataknál (Lyc. exsicc.) ; Bánfalva (Szent.); Répcze mellett Borsmonostor; Kapuvár Földsziget major; a Hanságban tömegesen. "
Gerlóczy 1907	[27]	-	1907	gyilkos csomorika		+	C, P, A	-	-	R, L, F	„mellyel a virágok vagy magvak rágicsálása útján gyermekeink mérgezhetik meg magukat; azután a *Cicuta virosát* vagy gyilkos csomorikát, a mely szép, fehér, ernyős virágaival, a zelleréhez hasonló leveleivel, a petrezselyemmel összetéveszthető gyökerével (mely azonban belül üreges) vagy az ánizshoz a csalódásig hasonló, de szagtalan gyümölcseivel már számtalan háztartási mérgezésre adott alkalmat, mert nemcsak a mocsaras helyeken, hanem a jól öntözött zöldséges-kertekben is felburjánzik és gyakran nem ismerik fel. Ennek a mérge a cikutoxin, mely szintén erős görcsöket okoz, bár más jellegűeket."
Hutyra 1908	[116]	-	1908	*Cicuta virosa*		-	-	against black cataract in cattle	-		„A fekete hályog leplezése könnyen sikerül az állat ügyes és gyors elővezetésével úgy a kocsi előtt, mint nyereg alatt. Ezért a vizsgálatot mindig az ügyben nem érdekelt egyének segédkezésével kell megejteni. Figyelmet érdemel továbbá, hogy némely idegmérgek (*Atropa belladonna, Hyoscyamus niger*, illetve ezek hatóanyagai, továbbá a cocain) tágítják a szem bogarát s az alkalmazkodás bénításával a látóképességet is gyöngítik."
Kóssa 1909	[123]	Hanság, Alföld	1909	Kónyi gyökér		-	-	medicinal herb	-		„Kónyigyökér. Csaplovics. (Gemaelde von Ungern, I. 74.) írja:"Bemerkenswerth ist eine Giftpflanze, welche im Hanság wie auch in den Sümpí'en Niederungerns haeufig angetroffen wird. Sie heisst Kónyi-Gyökér, Kónyer Wurzel voin Kónyer-Teiche, wo sie haeufig waeehst... Diese Wur zel ist nichts anders als der grosse Wasserschierling. (*Cicuta virosa*.)"
Margittai 1911	[125]	Kislónya	1911	*Cicuta virosa*	7699.4	-	-	-	-		„A sík terület ama része, mely a Tisza, a Szernye-csatorna, a Budapest—Lavocnei vonatrész, Kislónya és Mezőkaszony közt terül el. Ritkasag: *Cicuta virosa*."
Herman 1914	[115]	Puszta Angyalháza	1914	csomorika	8492.4	-	-	-	-		„Csomorika — P.-Angyalháza — *Cicuta virosa* L."Legmérgesebb fű, olyan fű, a melynek gubóját a pók összeszövi"
		Ecsedi láp	1914	mételytorzsa	8100.4	-	-	-	-		„Mételytorzsa — Ecsedi láp"
Reményik 1920	[54]	-	1920	gyilkos csomorika, vízi bürök, métely torzsa		-	-	Antidote: tannic acid	horse		„Gyilkos csomorika. Vízi bürök, métely torzsa. Évelő, megnő 0.7—1 m.-ig, vaskos, húsos tőkéje falakkal üregekre osztott. Levelei szorosan szárnyasak, fűrészesek. Virágzata összetett ernyős, ernyője 10—15 ágú, gallérja nincs, gallérkája sok levelű, szirom virágai fehérek. Virágzik lucius, augusztus. Az egész növény mérges, tartalmaz egy erős alkaloidát a Cicutint. Mérgezési esetek legelő állatoknál előfordulnak, a mérgezési tünetek hamar jelentkeznek. Kórtünetek: Étvágy hiány, nyálfolyás, hányás, kolika, a hátsótest felpuffadása, hasmenés, rendellenes érvelés és szívműködés, szembogár tágulás, szédülés. A halál rendesen egy fél óra 8. óra múlva bekövetkezik erős görcsök kíséretében fél kg. szárított csomorikától a lovak már elpusztulnak. Ellenszerek: Csersav, továbbá a gyomor kiürítése, tüneti kezelés."
Anonymus 1923	[57]	-	1923	csomorika		+	-	Abort.	-		„De lássuk meg azt is, hogy mikor a faluban letelepszünk, nem egyszer gyomoskertben járunk. Ott a kerítő, az ebszőlőnek személyesítője, ott a javasasszony, a foltos bürök, végre ott a magzatölő, a gyilkos csomorika. Ki kell irtani a gyomot, hogy tenyésszen az Isten termése."
Boros 1924	[102]	Kelemér	1924	*Cicuta virosa*	7688.4	-	-	-	-		„Im Moore sammelte ich noch folgende: Lophocolea heterophylla (Schrad.) Dum. var. nov. turfosa Schi ff n. (det. Schiff n.)3, Aulacomnium palustre (L.) Schwgr., Mnium Seligeri Jur., Polytrichum commune L., P. strictum Banks., Brachythecium Mildeanum Sch imp., Amblystegium riparium (L) B. E., Hypnum (Drepanocladus) Kneiffii (B. E.) Schi mp., H. (Campylium) chrysophyllum Brid., Calliergon cordifolium (Hedw.) Kindb., Nephrodium thelypteris, N. cristatum (L.) Mich., Carex rostrata, Juncus effusus, Salix cinerea, Betula pendula, Drosera rotundifolia, Epilobium palustre, Lythrum salicaria , Menyanthes trifoliata, Utricularia vulgaris."
Boros 1925	[103]	Veresegyházi-tó	1925	*Cicuta virosa*	8381.4	-	-	-	-		„Az úszó láp kialakulásához sok idő kell, ott tehát, ahol úszó láp van, rendesen érdekes növényzet is díszlik, például ilyenek a Veresegyházai- és Pósa-tó. Előbbi lápján az Alföldön ritka vidrafű (Menyanthes trifoliata), a Carex pseudocyperus nevű sás, mindkettőn a felette mérges csomorika (*Cicuta virosa*), a Posa lápján ezenkívül a tőzegharaszt és a lápi cserbóka (Sonchus palustris) is él."
		Pósa-tó	1925	*Cicuta virosa*	9277.1	-	-	-	-		„Az úszó láp kialakulásához sok idő kell, ott tehát, ahol úszó láp van, rendesen érdekes növényzet is díszlik, például ilyenek a Veresegyházai- és Pósa-tó. Előbbi lápján az Alföldön ritka vidrafű (Menyanthes trifoliata), a Carex pseudocyperus nevű sás, mindkettőn a felette mérges csomorika (*Cicuta virosa*), a Posa lápján ezenkívül a tőzegharaszt és a lápi cserbóka (Sonchus palustris) is él."
Darvas & Magyary-Kossa 1926	[39]	-	1926	*Cicuta virosa*		+	P	-	-	L	„Levelének petrezselyeméhez hasonló íze van, s így sokszor okozott mérgezéseket."
				Kónyi gyökér		-	-	against rheumatism	-	R	„Gyöktörzséből („kónyigyökér") régebben kivonatot készítettek a csúzos bajok ellen alkalmazták az orvosi gyakorlatban"
Pillich 1927	[134]	Simontornya	1927	*Cicuta virosa*	9277.1	-	-	-	-		„Így csak azon simontornyai vadon termő fajok, fajváltozatok és hybridek nevét sorolom fel a"Magyar flóra" beosztását követve, melyek eddig a megyei irodalomban említve nincsenek; ezek a következők: […] *Cicuta virosa* L. (leg. Boros)"
Pauer 1931	[133]	Szentkút alatt Kismákfa felé (Vas megye)	1931	gyilkos csomorika	8966.2	-	-	-	-		„A Szentkút alatt úgy 5 percnyire Kismákfa felé szintén még érintetlen ősmocsár terül el mézgás égerrel (Alnus glutinosa) és kevesebb számú magas kőrisfával (Fraxinus excelsior), továbbá mocsári gólyahírrel (Caltha palustris), gyilkos csomorikával (Cicuta virosa), mocsári kocsorddal (Peucedanum palustre), kesely derezsével (Selinum carvifolia), sással (Carex elongata), paizsikákkal (Nephrodium thelypteris és spinulosum), óriási zsurlóval (Equisetum maximum) stb. mint aljnövényzettel."
Nagy 1931	[130]	Debrecentől északkeletre	1931	vízi bürök, csomorika	-	-	-	-	-		„Ugyancsak Debrecentől északkeletre megtaláljuk a buckák közti laposokat is, amelyeknek virágos szőnyege a legmélyebb helyeken a feltörő talajvíz miatt nedves rétbe, sőt zsombékosba, avagy mocsárba megy át. … mérges vízi bürök vagy csomorika (*Cicuta*), nagy fehér ernyő virágzatával"
Anonymus 1932	[135]	-	1932	gyilkos csomorika		+	P	-	-		„Itt felsorolunk néhány növényt, amelyektől távol kell tartani a gyermekeket: dohány, foltos bürök, aranyeső, farkascseresznye maszlagot nadragulya, légyölő galóca. […] Vizes árkok partjain, mocsaras helyeken nyáron virágzik a gyilkos csomorika, amelynek íze és szaga hasonlít a petrezselyemre s ezért össze is lehet téveszteni a kettőt."
Kiss 1939	[120]	Sátoraljaújhely	1939	*Cicuta virosa*	7696.3	-	-	-	-		„*Cicuta virosa*… Sau: Longie"
		Sárospatak			7795.1	-	-	-	-		" *Cicuta virosa* … Sp: Bodrog holt ágaiban "
Missuray-Krúg 1943	[127]	Hanság, Rábaköz	1943	Kónyi gyökér		-	-	-	-		"A nép Hany Istókot nagyon sok esetben csak Csicsa"-nak nevezi még ma is. … Úgy elbódult a Hanyban, mint Csicsa a Kónyi gyökértől."'
Kiss 1943	[121]	Kis-Zsadány	18-19th *c.*	mételytorzsa	7996.2	-	-	-	cattle		„A heverő marhát kihajtották a Nagy Mága, Szilasoldal, Gégény, Bikaszigetre. … Kis Zsadányban mételytorzsát [kapott], amit ritkán hevert ki."
Réfi Oszkó 1997	[143]	Tiszarád	18-19th *c.*	mételytorzsa	7896.4	-	-	-	cattle		„Beszterec, Demecser, Tiszakanyár, Nagyhalász, Ontelek, Vasmegyer, Tiszarád lakói azért panaszkodtak, mert a rétek nyirkosak, a legelőkön mételytorzsa terem, s emiatt az állatok elpusztulnak."
		Vasmegyer			7896.4	-	-	-			
		Óntelek			7897.2	-	-	-			
		Nagyhalász			7896.1	-	-	-			
		Tiszakanyár			7797.4	-	-	-			
		Demecser			7897.1	-	-	-			
		Beszterec			7897.1	-	-	-			

Source	Ref. (1)	Location	Date	Name	Code (2)	Human pois. (3)	Misid. (4)	Ethnom.(5)	Anim. (6)	Plant part (7)	Original text
Földi in Nagy 1976	[29]	Hadház	18th *c.*	tsomorika, mételytorzsa	8396.1	-	-	-	sheep	R	„Nevezetes történet esett ezzel (t. i. a tsomorikával) ez előtt esztendővel … Egy nyáj juhot téli időben szénával éppen olyan rét aljon étettek, ahol az ilyen Tsomorikának torzsás gyökerei bőven találtattanak. Ezt a juhok még inkább rágták, mint a szénát (minthogy ezt édességének okáért különben is szeretik). De csakhamar kezdetiének is tőle számosan ledobbanni és megdögleni. A juhász és a gazdák keresik ezen történetnek okát és a legszembetűnőbb környülállás szerént a szénára vetnek. Egy oláh asszony oda megyén és azt mondja, hogy a juhok megették a Métely torzsát és attól döglenek. … Egy döglött juhot azonnal felbontatván, a gyomrában azon kimutatott Métely torzsa megtaláltatott. énnekem is hoztak belőle tudakozásnak okáért, hogy micsoda gyökér lehet az? és én azonnal megesmértem, hogy az a Tsomorika."
Frech in Gémes 1997	[40]	Kiskunmajsa	19-20th *c.*		9584.2	+	-	Abort.	-	R	"Kiskunmajsán (Bács-Kiskun m.) a nép abortív szerként használja; hosszú gyökerét megmosva, a méhszájba óvatosan feltolva 3 óra múlva abortuszt idézett elő (Frech’). Hasonló felhasználását ismerjük még Kígyósról és Szankról (Bács-Kiskun m.) (Frech’).”
		Szank			9484.1	+	-	Abort.	-	R	
		Kígyós			9584.4	+	-	Abort.	-	R	
Greschik 1939	[49]	-	-	csomorika		-	-	-	-		„Vannak madarak, melyek növényi mérgekkel szemben érzéketlenek. … Pacsirta és fürj veszély nélkül szedhetik a csomorika (*Cicuta*) magvait.”
Leopold 1924	[151]	Hanság	-	Kónyi gyökér		-	-	-	-		„Majd egy évszázada megújul a műszaki vesződség, rekeszték, zsilip, fő- és mellékcsatorna épül, már a Hanság szabad tavai összébb és összébb húzódnak … Hanem a születő hansági szárazföld még csak durva sásos, fakadásos rétet ad, … gyilkol a kónyi gyökér és legelői métely”
Márton 1968	[35]	Dévaványa - Cserepes	-	csomorika	8892.3	+	-	-	sheep	L	„… mérgező, úgyszintén a csomorika is, ennek a levele hasonlít a takarmányrépához, retek leveléhez. Egy asszony a riszeges urának csomorikalevelet főzött bele a paprikáshusba. Estére meghalt az ember, de kitudódott.”
Rácz 1988	[141]	Doboz	-	csomorika fű	9293.3	-	-	-	-		„csomorika fű"
